# Hypolipidemic and Antioxidant Properties of Phenolic Compound-Rich Extracts from White Ginseng (*Panax ginseng*) in Cholesterol-Fed Rabbits

**DOI:** 10.3390/molecules181012548

**Published:** 2013-10-10

**Authors:** Lan-Sook Lee, Chang-Won Cho, Hee-Do Hong, Young-Chul Lee, Ung-Kyu Choi, Young-Chan Kim

**Affiliations:** 1 Korea Food Research Institute, Seongnam, Kyonggi 463-746, Korea; E-Mails: sohee0809@hanmail.net (L.-S.L.); cwcho@kfri.re.kr (C.-W.C.); honghd@kfri.re.kr (H.-D.H.); yclee@kfri.re.kr (Y.-C.L.); 2 Department of Food Science & Technology, Korea National University of Transportation, Jeungpyeong 368-701, Korea; E-Mail: ukchoi@ut.ac.kr

**Keywords:** *Panax ginseng*, hyperlipidemia, antioxidant activity, atherosclerosis

## Abstract

In this study, the effect of low-molecular weight white ginseng compounds on various biochemical indices, including blood lipid concentrations and antioxidant enzyme activities and morphological changes was investigated in rabbits with high cholesterol diet-induced hypercholesterolemia. The experimental animals were 16-week-old male New Zealand white rabbits divided into normal control diet, high cholesterol diet, and high cholesterol with 0.05% white ginseng low-molecule compound groups, treated for 4 weeks. Blood lipid concentrations were higher in the high cholesterol groups compared to the normal control group but were not improved by the white ginseng low-molecular weight compound. We note however that antioxidant enzyme activities and morphological changes of the aorta showed that white ginseng small compounds had a positive effect on hypercholesterolemia. Based on such results, low-molecular weight compounds rich in phenolic compounds in white ginseng can be said to be effective in part in improving hyperlipidemia and atherosclerosis induced by a high cholesterol diet among New Zealand white rabbits.

## 1. Introduction

Cardiovascular diseases, which have been on the rise as an important health problem in modern society, are one of the main causes of death and are known to be induced mainly by atherosclerosis and triggered by general risk factors such as hypertension, hyperglycemia, smoking, and stress [1,2]. These risk factors are said to increase oxidative stress, which then causes various diseases such as cancer, heart disease, atherosclerosis, digestive disease, and autoimmune disease and aging [3,4,5,6]. Moreover, reactive oxygen species (ROS), oxygen compounds produced through general metabolic pathways in the body, are known to induce oxidative damages to proteins, fats, and DNA, hence the need to develop natural antioxidants rich in polyphenolics and phenylpropanoids. In particular, polyphenolic compounds, which are effective in the elimination of ROS by the action of their phenolic hydroxyl groups, have been considered very important in relation to antioxidant functions.

Ginseng, particularly the root of *Panax ginseng*, is mainly grown in Korea, China, and North America and has been used as a medicinal plant for a long time in the Orient. Depending on the manufacturing method, ginseng can be divided into white ginseng, which is naturally dried ginseng, and red ginseng, which is steam-dried ginseng [7]. Recently, the consumption of ginseng has increased greatly, even in Western society, and a variety of ginseng products have been developed and manufactured in more than 50 countries. Ginseng is known to affect various tissues including nervous, cardiovascular, endocrine, and immune system tissues; its major physiologically active ingredients include ginsenosides, polysaccharides, amino acids, polyacetylenes, alkaloids, and phenolic compounds [8]. A major class of active compounds related to the physiological activity of ginseng are the ginsenosides, which are divided into dammarane type and oleanane type, depending on the binding sugar moiety; research studies on ginseng have mainly studied the efficacy of saponins such as ginsenoside, although ginseng saponins have been reported to have relatively low antioxidant effects [9,10,11,12,13,14]. Compared to saponin compounds, studies on antioxidant activity using ginseng non-saponin compounds are very limited, and they include the antioxidant activity of white ginseng extracts prepared by enzyme treatment on V79-4 cells by our team [10] and an antioxidant study in mice of single compounds such as salicylic acid, vanillic acid, and *p*-coumaric acid found in ginseng [11]. Likewise, antioxidant activity has been known not to be expressed by a single ingredient, but rather a synergistic effect involving several compounds [15,16].

Thus, this study was conducted to investigate in rabbits fed with a high cholesterol diet the antioxidant activity of low-molecular weight compounds rich in phenolic compounds among the antioxidants extracted from white ginseng by a diethyl ether-ethyl acetate mixture and to test the physiological efficacy of non-saponin low-molecular weight compounds of ginseng.

## 2. Results and Discussion

### 2.1. Contents of Total Phenolic Compounds

The health benefit of medicinal plants usually comes from the antioxidant properties of phenolic compounds in the plant. There are various phenolic compounds in plants, ranging from simple polymerized substances to highly polymerized ones. In this study, low molecular weight phenolic compounds in white ginseng were extracted using diethyl ether and ethyl acetate with medium polarity. The amount of phenolic contents, expressed as g of gallic acid equivalents per 100 g of white ginseng was 1.2 g/100 g. Hwang *et al.* reported that the phenolic compounds in the ethyl acetate fractions of white ginseng were found to be 1.15% cinnamic acid, 0.19% *p*-coumaric acid and 0.24% quercetin by HPLC [17]. In general, phenolic compounds including vanillic acid, *p*-coumaric acid and ferulic acid have been reported to play an important role in the antioxidant activity of ginseng [9,10,11,12,13,14]. Especially, Liu *et al*. reported that saponin purified from ginseng has either no or only weak antioxidant activity, leading to the suggestion that phenolic compounds rather than saponins are responsible for ginseng’s antioxidant properties [9]. Kim *et al.* reported that ethyl acetate extracts of white ginseng have antioxidant activities on V79-4 cells [10]. Moreover, phenolic compounds, which are of lower molecular weight than ginsenosides Rg1, Re, and Rb1, have stronger antioxidant effects [14].

### 2.2. Effects on Body Weight Gain and Food Efficiency Ratio

The body weight gain and food efficiency ratio of male New Zealand white rabbits after 4 weeks of experimental diet consumption are presented in Table 1. The average weekly body weight gain and food efficiency ratio were lowest in the high cholesterol diet (Control) group, but the difference was not statistically significant. This is consistent with the results of Ramachandran *et al.* [18], who reported no significant difference in body weight gain between the normal diet group and high cholesterol diet group. Moreover, Matos *et al.* [19], Hartvigsen *et al.* [20], and Otunola *et al.* [21] all reported a significant decrease in body weight in the high cholesterol diet group, similar to this study.

**Table 1:** Body weight gain andfood efficiency ratio in the rabbits fed the experimental diets.

Group	Body weight gain (kg/4 weeks)	Food efficiency ratio
Normal	0.64 ± 0.18 ^NS^	0.12 ± 0.03
Control	0.41 ± 0.30	0.08 ± 0.06
Ginseng	0.57 ± 0.13	0.11 ± 0.03

All values are mean ± SD for 7 rabbits. NS; not significant. Food efficiency ratio; body weight gain/food intake. Normal diet; Control, high-cholesterol diet containing 0.05% white ginseng low-molecule extracts.

### 2.3. Effects on Plasma Lipid Profiles

Plasma lipid concentrations are shown in Table 2. Plasma TG was not significantly different among experimental groups, and total cholesterol and LDL cholesterol contents were considerably higher in the cholesterol-fed groups, Control group, and Ginseng group compared to the Normal group. In other words, a 1% high cholesterol-added diet induced 42~46 times higher hypercholesterolemia compared to Normal, but the feeding of low-molecular weight extract of white ginseng did not show any blood cholesterol-reducing effects. HDL-C content was not significantly different among groups but increased slightly with white ginseng low-molecular weight extract feeding. In general, the increase in total cholesterol in the blood is known as the abnormal index of lipid metabolism in the body such as coronary artery disease or problems in lipid metabolism, and LDL-C is said to be a risk factor for atherosclerosis and cardiovascular diseases due to its action on the accumulation of cholesterol in the arterial wall, causing the hardening of the artery. HDL (high-density lipoprotein) in the blood is also deemed to play the role of reverse cholesterol transport (RCT), transporting cholesterol from extra-hepatic tissues to the liver cholesterol; thus lowering the risk of cardiovascular disease [22]. According to Joo [23] and Yamamoto and Kumagai [24], ginseng saponin reduced the blood cholesterol concentration by increasing cholesterol secretion through bile acid synthesis. Yokozawa [25] reported that blood cholesterol content decreased by promoting LDL receptor synthesis, contrary to our results. On the other hand, Kang *et al.* [26] claimed that the administration of ginseng ginsenoside was not effective in lowering blood cholesterol in hypercholesterolemic rabbits, which was consistent with our results. In conclusion, the disagreement in the effect of ginseng on lowering cholesterol in the blood can be attributed to the composition of ginseng extract used in the experiments, dosages, and experimental periods.

**Table 2:** Plasma lipid profiles in the rabbits fed the experimental diets.

	**Group**		
	Normal	Control	Ginseng
Total triglycerides (mg/dL)	69.0 ± 6.6	128.3 ± 117.5	116.5 ± 59.9
Total cholesterol (mg/dL)	49.3 ± 12.9 ^b^	2090.6 ± 415.2 ^a^	2238.0 ± 502.0 ^a^
HDL-cholesterol (mg/dL)	23.6 ± 6.2	18.9 ± 3.6	21.5 ± 6.1
LDL-cholesterol (mg/dL)	15.4 ± 6.6 ^b^	762.9 ± 96.3 ^a^	771.5 ± 224.8 ^a^

All values are mean ± SD for 7 rabbits. Values within a row with different letters are significantly different by ANOVA with Duncan’s multiple range test at *p* < 0.05. Normal diet; Control, high cholesterol diet; Ginseng, high-cholesterol diet containing 0.05% white ginseng low-molecule extracts.

### 2.4. Effects on Hepatic Antioxidant Enzyme Activities

The hepatic tissues of experimental animals were collected after 4 weeks of experimental diet, and the antioxidant enzyme activities of GST, SOD, GPx, and CAT were analyzed, as shown in Table 3. CAT activity was significantly lower in the high-cholesterol diet groups compared to the Normal diet group (*p* < 0.05), with the ginseng low-molecular weight compound-added diet having no effect on CAT activity. SOD activity was significantly decreased by the high cholesterol-added diet compared to Normal, but the reduced activity by the high cholesterol-added diet was increased by about 41% by the ginseng low-molecular weight compound-added diet (*p* < 0.05). The amount of GSH and the activity of GST and GPx were not significantly different among experimental groups but tended to be higher in the Normal group. In the body, non-enzymatic anti-oxidation including GST and enzymatic anti-oxidation including GPx, CAT, and SOD occur to protect cell membranes and intracellular materials from reactive oxygen species, including free radicals. Generally, GSH is known to decrease in concentration by leaking outside the tissues with certain stimulation and is used as a substrate for GPx. SOD is a very important enzyme in the body produced in the early stage of free radical generation, converting superoxide into O_2_ and H_2_O_2_. GPx and CAT eliminate the H_2_O_2_ produced during the enzymatic reaction by SOD to protect the body from peroxidative damage. Kim and Park [27] reported that the activity of SOD and CAT increased by 31% and 24%, respectively, when ginseng extract was fed for 8 weeks to humans. Jin and Chang [28] stated that red ginseng extract fed to gamma-irradiated mice increased the activity of SOD, peroxidase, and CAT.

**Table 3:** Glutathione contents and hepatic antioxidant enzyme activities in the rabbits fed the experimental diets.

	**Group**		
	Normal	Control	Ginseng
GSH (mg/g liver)	21.1 ± 5.8	18.6 ± 4.4	16.7 ± 1.7
GST (unit/g protein/min)	1.5 ± 0.4	1.1 ± 0.3	1.3 ± 0.2
GPx (unit/mg protein/min)	21.8 ± 3.8	19.7 ± 3.0	19.8 ± 1.3
CAT (unit/g protein/min)	0.25 ± 0.03 ^a^	0.11 ± 0.01 ^b^	0.12 ± 0.03 ^b^
SOD (unit/g protein/min)	32.9 ± 10.2 ^a^	18.2 ± 5.1 ^c^	25.6 ± 8.2 ^b^

All values are mean ± SD for seven rabbits. Values within a row with different letters are significantly different by ANOVA with Duncan's multiple range test at *p* < 0.05. Normal diet; Control, high cholesterol diet; Ginseng, white ginseng low-molecule extracts in high cholesterol diet.

### 2.5. Effects on Plasma and Hepatic Lipid Peroxidation

**Figure 1:**
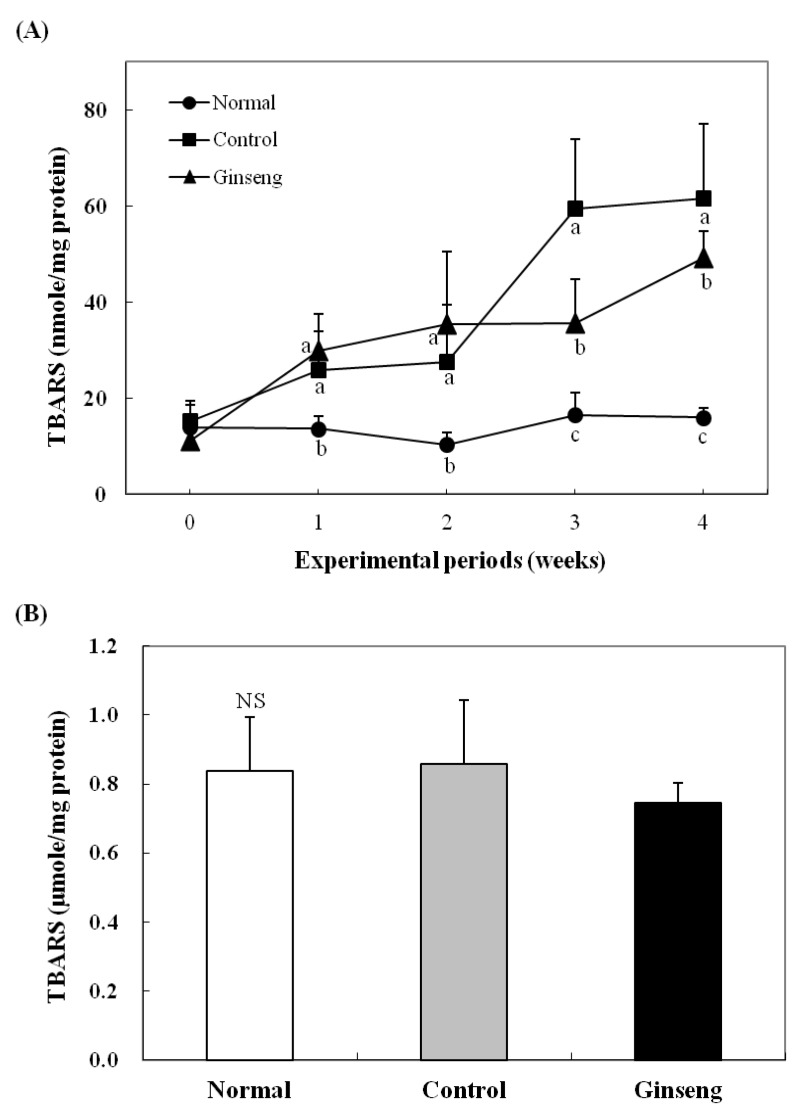
Plasma (**A**) and hepatic (**B**) TBARS level in the rabbits fed the experimental diets. All values are mean ± SD for 7 rabbits. Values with different letters are significantly different by ANOVA with Duncan’s multiple range test at *p* < 0.05 (NS; not significant). Normal diet; Control, high cholesterol diet; Ginseng, high-cholesterol diet containing 0.05% white ginseng low-molecule extracts.

To examine the effect of ginseng low-molecular weight compounds on the changes in lipid peroxides in rabbits under oxidative stress caused by a high cholesterol diet, the MDA content in the plasma and in the liver was measured, as shown in Figure 1. The weekly measured plasma MDA was not significantly different among experimental groups until 2 weeks but was significantly higher in the high cholesterol diet group (*p* < 0.05) after 2 weeks. On the other hand, the diet with added white ginseng low-molecular weight compounds suppressed MDA production by about 20% (Figure 1A). The liver MDA level after 4 weeks of experiment was not significantly different among experimental groups, but MDA production was reduced by about 21% in the Ginseng diet group (Figure 1B). The effect of ginseng low-molecular weight compounds in the diet on the reduction of MDA in the plasma and in the liver is considered the action of phenolic compounds in the ginseng extract, increasing the activity of antioxidant enzymes. According to Zang *et al.* [29], the water extract of ginseng inhibited the *in vitro* lipid peroxidation. Bak *et al.* [30] reported that the essential oil extracted by supercritical CO_2_ from the byproduct after the water extraction of red ginseng inhibited hepatic lipid peroxidation in mice with carbon tetrachloride-induced liver damage.

### 2.6. Morphological Changes of Aorta

**Figure 2:**
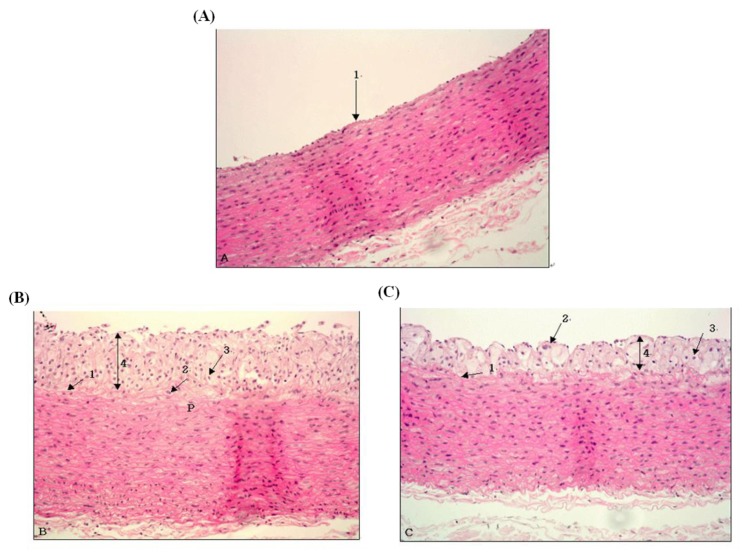
The aortic cross-section in the rabbits fed the experimental diets. (**A**): normal diet (**B**): high cholesterol diet (**C**): high-cholesterol diet containing 0.05% white ginseng low-molecule extracts (Magnification = 200×). * Note the thickening of intima and media due to edema and atheromatous plaque in the thoracic aorta in (B). 1: internal elastic lamina; 2: macrophage; 3: foam cell; 4: atheromatous plaque. ** Thickening of intima resulting from formation of atheromatous plaque was considerably attenuated (**C**). 1: internal elastic lamina; 2: macrophage; 3: foam cell; 4: atheromatous plaque.

**Figure 3:**
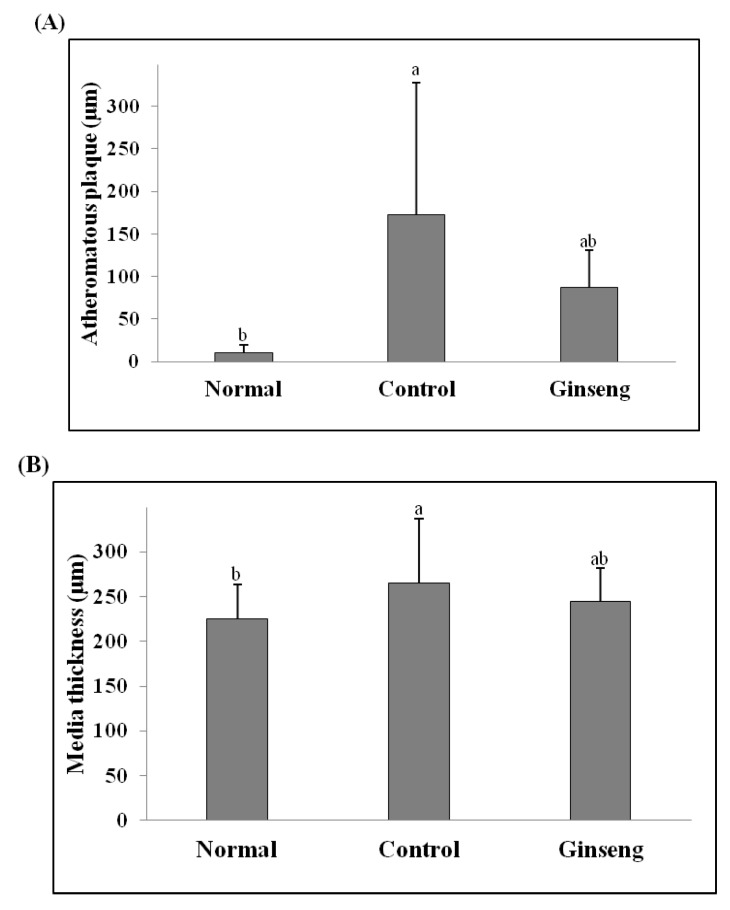
Atheromatous plaque (**A**) and Media thickness (**B**) in the rabbits fed the experimental diets. All values are mean ± SD for 7 rabbits. Values with different letters are significantly different by ANOVA with Duncan’s multiple range test at *p* < 0.05. Normal diet; Control, high cholesterol diet; Ginseng, high-cholesterol diet containing 0.05% white ginseng low-molecule extracts.

Figure 2 and Figure 3 present the histopathological results from the slices of the aorta in experimental animals after staining with H&E. The aortic wall in the Normal group was well maintained with neither damage nor pathological findings (Figure 2A). In the aorta of the high-cholesterol diet groups, the elastic lamina layer was greatly damaged, and macrophages and foam cells were found; arteriosclerosis progressed with the atheromatous plaque being as thick as 172.9 µm (Figure 2B). Figure 2C shows that, in the aorta in the ginseng low-molecule compound-added diet group, macrophages and foam cells were found as in the case of Control, but the thickness of the atheromatous plaque was 86.7 µm, showing that ginseng could reduce the thickness of the high cholesterol diet-induced atheromatous plaque by about 50% (Figure 3A). The thickness of the media was significantly lowest in the Normal group at 225 µm (*p* < 0.05) and significantly highest in the high cholesterol added Control group at 265 µm (*p* < 0.05) (Figure 3B). In general, the most important causes of atherosclerosis have been known to be hypercholesterolemia and increased LDL cholesterol in the blood; risk factors promoting atherosclerosis include hypertension, smoking, stress, and aging [1,2]. The generally known developmental theory of atherosclerosis is “response to injury hypothesis” described by Ross [31], wherein hyperlipidemia and toxic substances cause endothelial cell damage and proliferation, including the movement of monocytes inside the blood vessel walls and transformation into macrophages, accumulation of lipids and dead cell particles, smooth muscle cell proliferation and fibrosis, platelet adsorption and aggregation and subsequent vasoconstriction and angiostenosis, and thickening and narrowing of blood vessels and hardening of the arterial wall. In this study, macrophages and foam cells were largely found after a high cholesterol diet, with the progression of atherosclerosis, including the thickening of the aortic wall, being observed. We note, however, that the addition of white ginseng low-molecular weight compounds to the diet greatly improved the symptoms of atherosclerosis.

## 3. Experimental

### 3.1. Preparation of White Ginseng Extracts

White ginseng used in the experiment was purchased at a ginseng market in Geumsan, Republic of Korea and pulverized to average particle size of 100 µm by a pulverizer (Blender HGB7WTS3, Waring Commercial, Torrington, CT, USA). Low molecular weight phenolic extracts were prepared by the method described by Krygier *et al.* with some modifications [32]. White ginseng powder was extracted with methanol–acetone–water (6:6:4, *v/v/v*) at room temperature. After centrifugation, the supernatant was collected and the pellet was re-extracted two additional times. The combined supernatant was evaporated to remove methanol and acetone and the low molecular weight phenolic compounds were extracted twice with diethyl ether-ethyl acetate (1/1, *v/v*) in a shaking incubator (SIF-6000R, Jeio Tech. Co., Ltd., Daejeon, Korea). The organic extracts were then concentrated using a rotary evaporator (R-114, Buchi Labortechnik AG, Flawil, Switzerland) and freeze-dried (MCFD series, IlshinBioBase, Yangju, Korea) as samples for the experiments.

### 3.2. Determination of Phenolic Contents

Phenolic contents in white ginseng extracts were measured by the modified Folin-Ciocalteu assay carried out according to the method described by Singleton and Rossi [33]. The extracts (0.1 mL) were redissolved in 50% aqueous methanol and mixed with 0.2 N Folin-Ciocalteu reagents (1 mL). The mixture was allowed to stand at room temperature for 3 min after which saturated sodium carbonate (1 mL) was added. On incubation at room temperature for 60 min, the absorbance was recorded at 735 nm. Phenolic contents were calculated by comparing with an external standard calibration curve of gallic acid and were expressed as gallic acid equivalents (GAE, g gallic acid) per 100 g of white ginseng.

### 3.3. Animals and Diets

A total of 21 male 16 weeks old New Zealand white rabbits were purchased from Central Lab-Animal, Inc. (Seoul, Korea) and acclimated on a normal diet (Purina, Seoul, Korea) which consisted of 16% protein, 3.5% vegetable fat, 18.5 to 21.5% fiber, 0.7 to 1.2% calcium, 0.4% phosphorus and 0.5 to 1.0% sodium chloride for one week prior to experimentation. After this, experimental groups were divided into normal control diet (Normal), high-cholesterol diet (Control), and high-cholesterol diet containing 0.05% white ginseng low-molecule extracts (Ginseng); seven rabbits were assigned to each group by randomized block design. Hypercholesterolemic diet was prepared by adding 1% (w/w) cholesterol (Sigma, St. Louis, MO, USA) to normal diet. The temperature and humidity of the breeding room were maintained at 19 ± 1 °C and 55 ± 5%, respectively, with 12-hour light-dark cycle. Diets were provided at 100 g/kg body weight, with the body weight measured once a week during the experimental period. Blood was drawn from the posterior auricular vein once a week for the measurement of antioxidant activity. The breeding and management of all experimental animals followed the IACUC regulations.

### 3.4. Collection of Blood and Organs

After 4 weeks of experimental diets, animals were fasted for 16 h, and blood was drawn from the retro-orbital plexus for blood analysis. For the separation of the serum, the blood was left at room temperature for 30 min and centrifuged at 4 °C, 1,000 g for 15 min (5810R, Eppendorf AG, Hamburg, Germany) to collect the supernatant. The liver was dissected and rinsed in physiological saline, blotted on filter paper, and weighed. The abdominal aorta including the aortic arch was dissected and rinsed in physiological saline and fixed in 10% neutral formalin duffer. All samples were stored in a freezer at −70 °C for further physicochemical experiments.

### 3.5. Measurement of Blood Lipids

Plasma concentrations of triglyceride (TG), total cholesterol (TC), HDL-cholesterol (HDL-C), and LDL-cholesterol (LDL-C) were measured using a quantification kit (Bayer, New York, NY, USA) by automatic chemistry analyzer (Hitachi 7180, Hitachi, Tokyo, Japan).

### 3.6. Measurement of Glutathione Content and Enzyme Activity

#### 3.6.1. Liver Preparation for Enzyme Assay

An ice-cold 0.1 M PBS (pH 7.4) solution was added to liver tissues and homogenized using a homogenizer (Daihan, Seoul, Korea). Part of the homogenate was used for malonaldehyde (MDA) measurement, with the remaining part centrifuged at 10,500 g for 1 h and the supernatant used for enzyme source for the measurement of antioxidant enzyme activity.

#### 3.6.2. Glutathione (GSH) Content and Glutathione S-Transferase (GST) Activity

The amount of GSH was measured using the method of Hissin and Half [34]. Phosphate-EDTA buffer (pH 8.0, 4.5 mL) was added to supernatant (0.5 mL). The final assay mixture (2.0 mL) contained 100 μL of the diluted sample, 1.8 mL phosphate-EDTA buffer, and 100 μL *o*-phthalaldehyde (OPT) solution. After mixing and incubation at room temperature for 15 min, the solution was transferred to a quartz cuvette. Fluorescence at 420 nm was determined with activation at 350 nm (Varian, Walnut Creek, CA, USA). GST activity using 1-chloro-2,4-dinitrobenzene (CDNB) as a substrate was measured according to the according to the modified method of Habig *et al.* [35]. The reaction mixture consisted of 10 mM CDNB, 10 mM GSH, 0.2 M potassium phosphate buffer (pH 6.5), and sample. The change in the absorbance was measured at 340 nm for 3 min.

#### 3.6.3. Superoxidase Dismutase (SOD) Activity

The activity of SOD was measured according to the modified method of Carrillo *et al.* [36] based on the inhibition of cytochrome c by SOD. One hindred μL of the sample was added to 0.05 M pH 7.4 phosphate buffer (2 mL) containing 100 μM EDTA, 100 μM xanthine, 40 μM cytochrome c, and 0.01 units of xanthine oxidase. The reaction was initiated by adding xanthine oxidase, and then incubated at 30 °C for 3 min. The absorbance was measured at 550 nm. One enzyme unit was defined as the amount of enzyme required to inhibit cytochrome c reduction by 50%.

#### 3.6.4. Glutathione Peroxidase (GPx) Activity

GPx activity was measured according to the modified method of Wendel [37]. The reaction mixture contained 0.25 M phosphate buffer (pH 7.0, 0.6 mL), 10 mM GSH (0.3 mL), 10 mM EDTA (0.3 mL), 10 mM sodium azide (0.3 mL), 2 mM NADPH (0.3 mL), and glutathione reductase (20 μL). The mixture was added to the sample (0.9 mL) and incubated at 30 °C for 5 min. The reaction was initiated by the addition of 2.5 mM hydrogen peroxide (0.3 mL). The absorbance was measured at 340 nm for 2 min.

#### 3.6.5. Catalase (CAT) Activity

The activity of CAT was measured according to the modified method of Carrillo, *et al.* [36]. A mixture of 0.05 M sodium phosphate buffer (pH 7.4), 1 μM H_2_O_2_, and sample was made up to a final volume of 3 mL, and the decrease in absorbance was measured at 240 nm in 1 min. One unit of CAT activity was defined as the amount of enzyme required to decompose 1 μM of H_2_O_2_ for 1 min.

### 3.7. Lipid Peroxidation in Liver

Malondialdehyde (MDA) levels, an index of lipid peroxidation, were measured by monitoring the thiobarbituric acid reactive substances (TBARS) according to the modified method of Ohkawa, *et al.* [38]. The reaction mixture contained sample (0.2 mL), 8.1% sodium dodecyl sulfate (0.2 mL), acetic acid (1.5 mL), and 0.5% TBA (1.5 mL). The mixture was heated in a water bath at 95 °C for 60 min. After cooling, *n*-butanol/pyridine (5 mL, 15:1, v/v) was added, followed by vigorous shaking. The mixture was centrifuged at 1,700 g for 10 min, and then the absorbance of supernatant (organic extract) was measured at 532 nm.

### 3.8. Histopathological Examination

Liver tissues and aorta were clipped and fixed in 10% neutral formalin duffer, embedded in paraffin, and then thinly sliced into 4 µm thick. They were stained with hematoxylin-eosin for slide preparation, and then observed for pathological abnormality using a light microscope.

### 3.9. Statistical Analysis

All experimental data were expressed as mean and standard deviation and tested by one-way ANOVA using the SAS program (SAS Institute, Cary, NC, USA), and the differences between the means were performed using Duncan's multiple range test at *p* < 0.05.

## 4. Conclusions

In the present study, effects of white ginseng phenolic compound-rich extracts on various risk factors of atherosclerosis and development of atherosclerosis in hypercholesterolemic rabbit have investigated. Blood lipid concentrations were not improved by the white ginseng extracts, but antioxidant enzyme activities and morphological changes of the aorta showed that white ginseng phenolic compound had a positive effect on hypercholesterolemia. These results suggest that diet-induced hypercholesterolemic atherosclerosis is associated with an increase in the oxidative stress and that white ginseng phenolic compound-rich extracts reduced the extent of atherosclerosis by improving oxidative stress.
